# Assessing the impact of occlusal plane rotation on facial aesthetics in orthodontic treatment: a machine learning approach

**DOI:** 10.1186/s12903-023-03817-y

**Published:** 2024-01-06

**Authors:** Jingyi Cai, Ziyang Min, Yudi Deng, Dian Jing, Zhihe Zhao

**Affiliations:** 1 State Key Laboratory of Oral Diseases & National Clinical Research Center for Oral Diseases, West China Hospital of Stomatology, Sichuan University, No.14, 3rd Section, South Renmin Road, Chengdu, Sichuan 610041 China; 2 Department of Orthodontics, Shanghai Ninth People’s Hospital, Shanghai Jiao Tong University School of Medicine; College of Stomatology, Shanghai Jiao Tong University, No.639, Zhizaoju Road, Huangpu District, Shanghai, 200011 China

**Keywords:** Orthodontics, Occlusal plane, Machine learning, Back-propagation artificial neural network, Aesthetic improvement

## Abstract

**Background:**

Adequate occlusal plane (OP) rotation through orthodontic therapy enables satisfying profile improvements for patients who are disturbed by their maxillomandibular imbalance but reluctant to surgery. The study aims to quantify profile improvements that OP rotation could produce in orthodontic treatment and whether the efficacy differs among skeletal types via machine learning.

**Materials and methods:**

Cephalometric radiographs of 903 patients were marked and analyzed by trained orthodontists with assistance of Uceph, a commercial software which use artificial intelligence to perform the cephalometrics analysis. Back-propagation artificial neural network (BP-ANN) models were then trained based on collected samples to fit the relationship among maxillomandibular structural indicators, SN-OP and P-A Face Height ratio (FHR), Facial Angle (FA). After corroborating the precision and reliability of the models by T-test and Bland-Altman analysis, simulation strategy and matrix computation were combined to predict the consequent changes of FHR, FA to OP rotation. Linear regression and statistical approaches were then applied for coefficient calculation and differences comparison.

**Results:**

The regression scores calculating the similarity between predicted and true values reached 0.916 and 0.908 in FHR, FA models respectively, and almost all pairs were in 95% CI of Bland-Altman analysis, confirming the effectiveness of our models. Matrix simulation was used to ascertain the efficacy of OP control in aesthetic improvements. Intriguingly, though FHR change rate appeared to be constant across groups, in FA models, hypodivergent group displayed more sensitive changes to SN-OP than normodivergent, hypodivergent group, and Class III group significantly showed larger changes than Class I and II.

**Conclusions:**

Rotation of OP could yield differently to facial aesthetic improvements as more efficient in hypodivergent groups vertically and Class III groups sagittally.

## Background

Aesthetic improvements constitute a major goal of contemporary orthodontics which emphasizes both dental and facial harmony. Patients who are dissatisfied with their profiles owing to maxillomandibular imbalance may benefit from orthognathic surgery, orthodontic camouflage, and orthopedic treatment, depending on their growth phase and the severity of the deformity. For adults who have passed the growth spurt phase but show mild-to-moderate deformity, successful orthodontic treatment could also contribute to impressive profile improvements by dentition alignment and adequate mandible rotation [1]. Though mandible growth could hardly happen for adults, the effect of beneficial rotation would not be underestimated in correcting both vertical and sagittal problems, as a counterclockwise rotation could compensate the deficiency of mandible as well as decrease the frontal face height, and vice versa [1–3].

As for the regulatory mechanism of orthodontics-induced rotation, studies have pointed out that alteration of occlusal plane (OP) which leads to subsequent mandibular rotation could be the key. First, from the standpoint of physiological growth, it is hypothesized that a continuous horizontalization of OP during mandibular growth is accompanied by a concomitant reduction of mandibular plane cant. Otherwise, aberrant changes in OP might influence the skeletal frame, resulting in malocclusions [4, 5]. The physiological reference value of OP-SN in mixed and permanent dentition is about 16.4 ° and 12.4 °, respectively. Nevertheless, imbalance grow as hypodivergent and hyperdivergent skeletal types could lead to nearly 6° divergence of SN-OP [5]. Sagittally, OP was statistically significantly steeper in Class II subjects compared to Class I and III [6]. Thus, a healthy occlusion and normal OP angle could be vital for skeletal development. Second, OP controlling plays an essential part during orthodontic treatment. Reducing OP angle in hyperdivergent Class II patients accompanied by mandibular counterclockwise rotation could be conductive to profile improvement and pharyngeal airway expansion [2, 7], and beneficial outcomes could be obtained as increase of OP angle and clockwise rotation of mandible for hypodivergent Class III patients [8]. Generally, there are three definitions of occlusal plane, bisected occlusal plane (BOP) by Downs [9], functional occlusal plane (FOP) by Wits [10], and an MM (maxillary-mandibular) bisector by Hall-Scott [11]. Considering the operability of cephalometric OP measurement, the definition of BOP would be used in this study considering it was suggested as a more reproducible reference plane during cephalometric tracing process [6].

To assess the improvement of profile, P-A face height and facial angle NP-FH could be suitable indicators for vertical and sagittal changes, respectively. P-A face height equals the ratio of S(sella)-Go(gonion) / N(nasion)-Me(menton), representing the vertical balance between anterior and posterior face height. When the value of P-A face height falls in the normative range of 60 ~ 65%, it usually indicates a matching growth pattern of anterior and posterior skeletal as well as dental tissues [12]. On the opposite, disproportion due to growth discrepancy or inadequate rotation of mandible could lead to some visual disharmony as long or short face, which could be a principal complaint of patients looking for orthodontic therapy. Another aesthetic factor that urges the request of orthodontics is the convexity of profile [13]. In cephalometric analysis, the facial angle NP-FH serves a role in reflecting the degree of mandibular protrusion. Axiomatically, neither anterior nor posterior position outside the normative range of mandible could result in an unpleasing profile as retrognathism or prognathism [14].

In regard to the two main aesthetic concerns, studies abound favoring the conductive roles of SN-OP changes to mandible rotation and profile improvements [2, 6, 15]. Nevertheless, there is no study suggesting the quantitative effects as profile improvements that SN-OP changes could produce and whether it differs among various skeletal growth pattern. The obstacle for solving above question mainly exists in the complex and non-linear relationship among cranium, maxilla and mandible structure, which contributes to a task intractable for traditional mathematics analysis and requires mass data analysis. Therefore, further research is needed to tease apart the interdependent and interactive geometrical relationships among occlusion and skeletal structure, and provide a theoretical basement for OP angulation controlling for therapeutical improvements.

Remarkably, with the booming progression of computer technology, machine learning has gained reputation for its characteristics as self-adaptive, self-learning and high accuracy, which could not only outweigh traditional tactics by its effectiveness, but also be competent in tackling down those so-called impossible tasks for its integration of both advantageous sides of human brain and computers [16, 17]. Among all the branch disciplines, the back-propagation artificial neural network model (BP-ANN) is a multi-layer feedforward neural network which could be trained to fit complex mathematical function containing various predictors [18]. We thus utilized the BP-ANN model to predict the profile improvements induced by OP changes under specific skeletal parameters. By taking advantage of the modeling ability of AI with plenty inputs and non-linear problems, we hope to provide orthodontists a quantitative evidence of the efficiency of SN-OP changes as a reference.

## Materials and methods

### Subjects

Cephalometric radiographs of patients visited West China Stomatology Hospital from January, 1st, 2020 to July, 1st, 2021 for orthodontic treatments were selected for dataset construction. We mainly included data of 18–35 years old young adults without congenital deformity, infection, trauma, tumor, surgery history. Meanwhile, patients with obvious skeletal malformation with indication of surgery were excluded to ensure the applicability of our network. Lateral cephalometry were obtained from a Cephalometer (Veraviewepocs, Morita, Kyoto, Japan) when subject was positioned with the Frankfort plane paralleled to the horizontal line with their teeth in centric occlusion and lips lightly closed.

### Cephalometric analysis

**Fig. 1:**
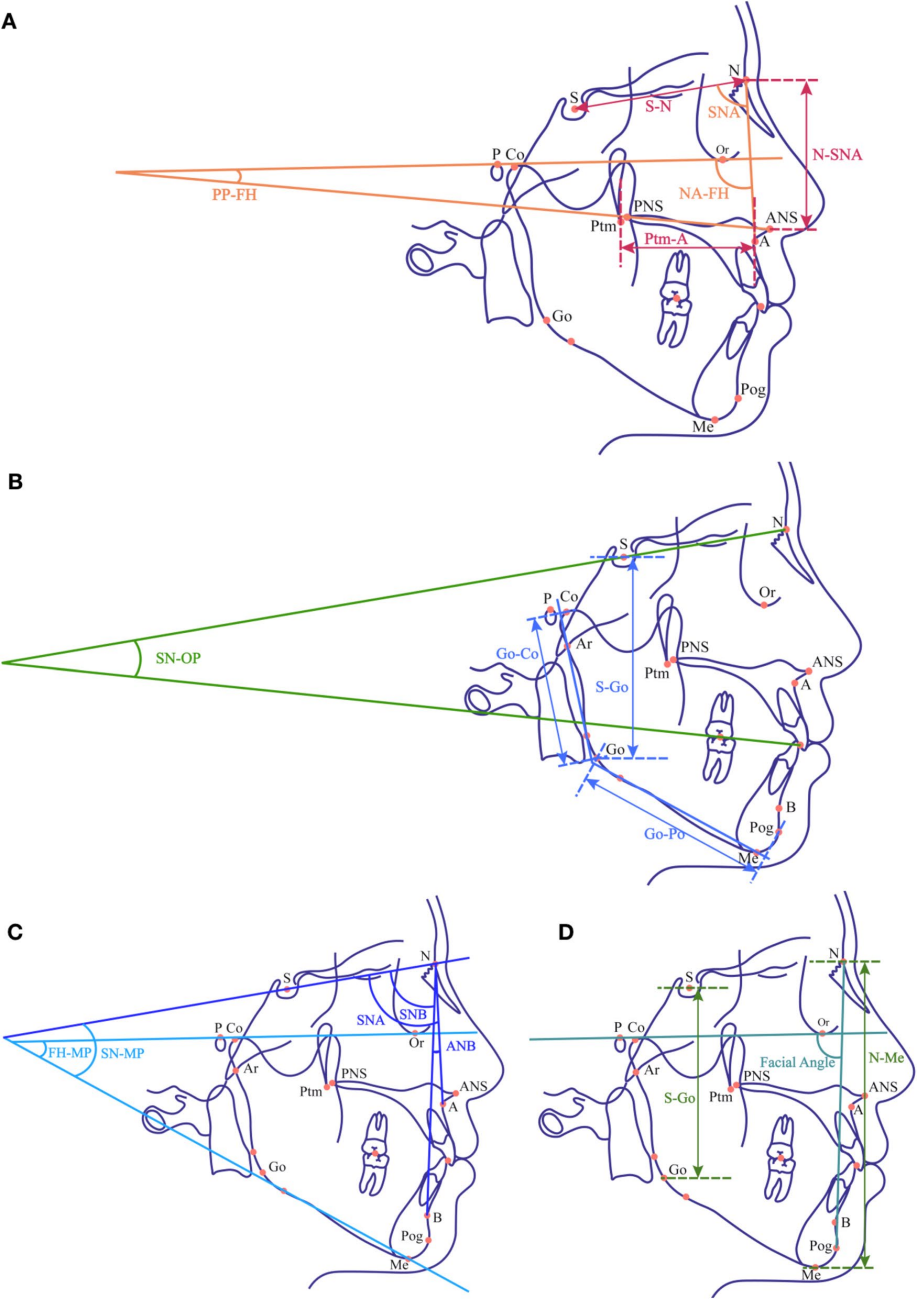
Schematic illustration of the landmarks and parameters used in the work. Detailed descriptions correspond to Table 1. **A** Variables representing cranial and maxillary skeletal structure. **B** Variables representing mandibular structure and SN-OP. **C** Parameters for sagittal and vertical group classification. **D **Predict targets as S-Go/N-Me (P-A face height%, FHR) and facial angel (FA)

The automatic cephalometric landmarks localization function of Uceph (China) was adopted to label the lateral cephalometry and two trained orthodontists manually checked and revised the marks. The landmarks and cephalometric measurements were illustrated by Fig. 1 and Table 1. Patients were classified by sagittal and vertical skeletal type based on criteria listed in Table 2. The Intra-class Correlation Coefficient (ICC) was used to assess inter-operator and intra-operator reliabilities. Briefly, 50 cephalograms were randomly selected and measured by three orthodontists. Each orthodontists repeated the measurements after 2 weeks. Both the inter- and intra-operator agreements of measurement were pretty good, indicated by the ICC scores of 15 parameters ranged from 0.90 to 0.98 and 0.88 to 0.94.

**Table 1:** Cephalometric indicators used in this study

Abbr.	Scale	Name	Definitions
**Crania and Maxilla variables**			
N-ANS	mm	Upper Facial Height	Vertical linear distance from the Nasion to Anterior Nasal Spine
S-N	mm	Extent of Anterior Cranial Base	Linear distance from the Sella to Nasion
Ptm-A	mm	Mandibular base length	Distance between pedals of perpendiculars of Go and Po to the Palatal Plane
SNA	deg	Sella-Nasion-A point Angle	A point angulation to Sella-Nasion
NA-FH	deg	Horizontal position of the maxilla relative to the cranial base	Angulation between the Sella-Nasion plane and the Frankfort Plane
PP-FH	deg	Palatal Plane to Frankfort Plane Angle	Angulation between Palatal Plane to the Frankfort Plane
**Mandible variables**			
Go-Po	mm	Extent of Mandibular Plane	Distance between pedals of perpendiculars of Go and Po to the MP
Go-Co	mm	Extent of Mandibular Ramus	Distance between pedals of perpendiculars of Go and Co to the Ramal Plane
S-Go	mm	Posterior Facial Height	Vertical linear distance from the Sella to Gonion
SN-OP	deg	Sella-Nasion Plane to Occlusal Plane Angle	Angulation between the Sella-Nasion plane and the Occlusal Plane
**Classification and Outcome Indicators**			
S-Go/N-Me	%	P-A Face Height	Ratio of the vertical linear distance of Sella- Gonion to the vertical linear distance of Nasion-Menton
NPo-FH	deg	Facial Angle	Angulation between Nasion- Pogonion and the Frankfort Plane
SNB	deg	Sella-Nasion-B point Angle	B point angulation to Sella-Nasion
ANB	deg	A point-Nasion-B point Angle	Angulation formed by A point, Nasion, and B point
FH-MP	deg	FMA (Frankfort Mandibular-Plane Angle)	Angulation between Frankfort Plane and the Mandibular Plane
SN-MP	deg	SN Plane to Mandibular Plane Angle	Angulation between the Sella-Nasion plane and the Mandibular Plane

**Table 2:** Classification criteria of each skeletal types

**Sagittal Classification**		
**Class I**	**Class I**	**Class I**
1° ≤ ANB ≤ 5°	1° ≤ ANB ≤ 5°	1° ≤ ANB ≤ 5°
**Vertical classification**		
**Hypodivergent**	**Normodivergent**	**Hyperdivergent**
Conform to 2 or more items: SN-MP < 24°; FH-MP < 22°; P-A face height < 62%	Conform to 2 or more items: 24 ≤ SN-MP ≤ 36°; 22 ≤ FH-MP ≤30°; 62% ≤ P-A face height ≤ 65%	Conform to 2 or more items: SN-MP > 36°; FH-MP > 30°; P-A face height > 65%

###  BP neural net fitting

**Fig. 2:**
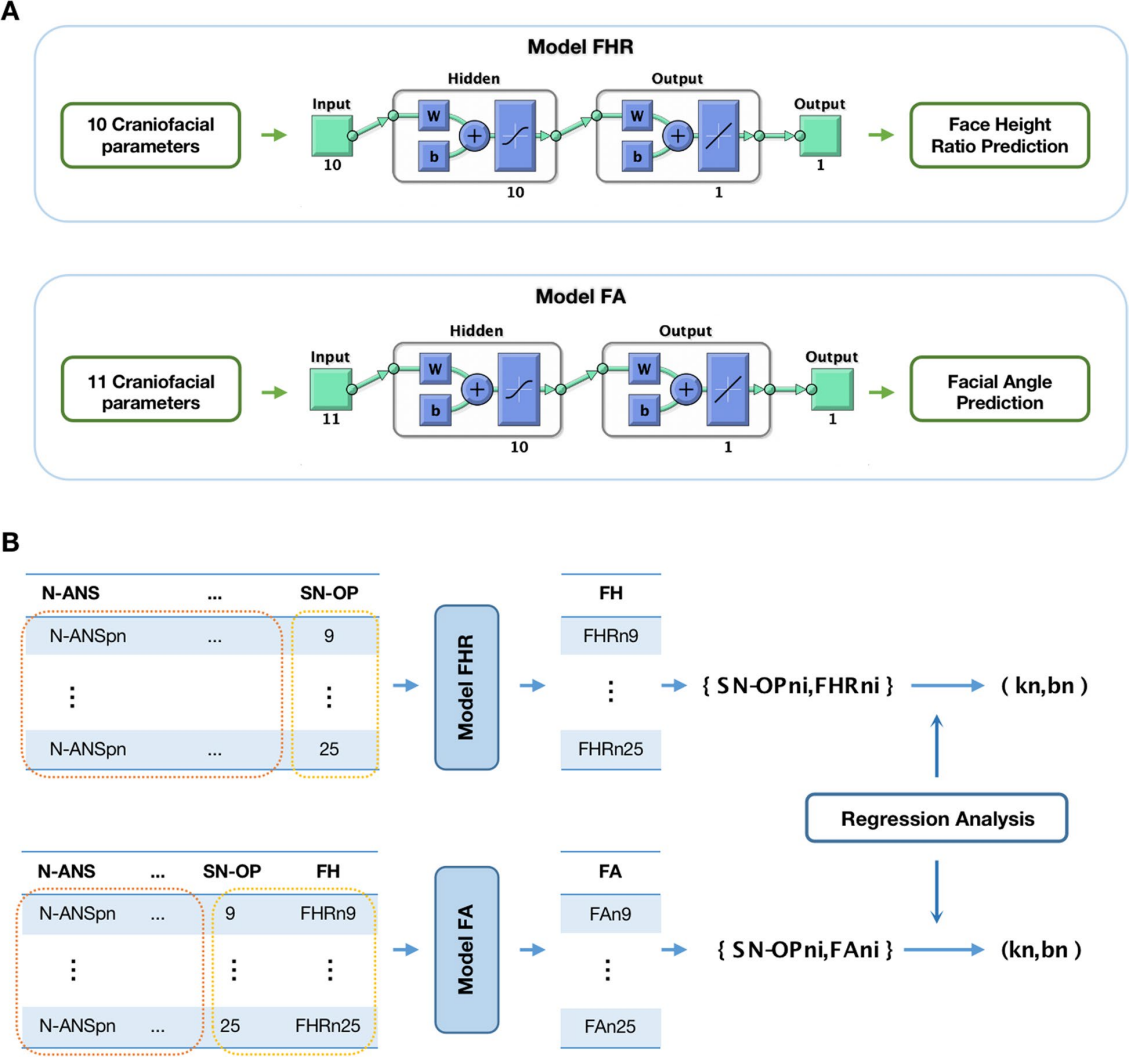
Model structures and workflow. **A** The structure of BP-ANN models. **B** The workflow to calculate the coefficients between SN-OP and FHR, FA respectively. Briefly, for FHR, patient-specific skeletal parameters (pn) were extracted to form a matrix where SN-OP varied from 9 to 25 (yellow box) while other variables as skeletal parameters remained constant (N-ANS, S-N, SNA, NA-FH, Ptm-A, PP-FH for cranio-maxilla complex, Go-Po, Go-Co, S-Go for mandible) (orange box). The matrix was put into the model FHR to predict patient-specific FHR values (FHRn9-FHRn25) in response to SN-OP changes. Subsequently, a regression function between SN-OPni and FHRni yielded slope (kn) and vertical intercepts (bn) for each patient. The FA model followed a similar process

Inputs were chosen from skeletal measurement parameters as N-ANS, S-N, SNA, NA-FH, Ptm-A, PP-FH for cranio-maxilla complex, Go-Po, Go-Co, S-Go for mandible, and SN-OP (Table 1). Noticeably, the input except SN-OP stand as the indicators of basic anatomic structure of cranium, maxilla and mandible, which could largely remain constant during orthodontic therapies regardless of mandibular rotation (Fig. 1.A, B). By excluding the parameters which have overlapping function with SN-OP in representing the mandible rotation, we separated the invariables into two main parts as: a) constant ones representing basic craniofacial structure, b) SN-OP standing for maxillo-mandible relationship and occlusion rotation. By this means, we ensured that alternation of SN-OP in the mathematical function was independent with other factors and represented the relative position of mandible. The corresponding values of P-A Face Height ratio (%) (FHR) and FH-NPo (Facial Angle, FA) were used as target, representing the esthetic improvement vertically and sagittally, individually. Variables were then all normalized to the range from − 1 to 1 by the mapminmax function of MATLAB. A three-layer feedforward BP-ANN model was built by MATLAB R2018a neural network toolbox. Samples were divided randomly into training, validation and testing dataset with a proportion of 75:15:15. The number of hidden layer nodes was set to 10 and the topological structure of the model was shown in Fig. 2A.

### Prediction of P-A face height (%) and FH-NPo (facial angle) correlation to SN-OP

To predict the correlation between P-A Face Height (%) and FH-NPo (Facial Angle) with SN-OP, we conducted simulations using our dataset. Specifically, we varied the SN-OP angle within a range of 9 to 25 degrees while keeping all other fundamental craniofacial parameters constant for each patient, resulting in an individualized input matrix for each case (Fig. 2). Using this simulated input matrix, which combined the patient’s baseline anatomical information with the altered SN-OP value, we employed model FHR to predict the corresponding FHR values across various SN-OP angles for each patient. Subsequently, we conducted a linear regression analysis to quantify the correlation between input SN-OP and out FHR. This analysis allowed us to calculate both the vertical intercepts (b) and slopes (k). In interpretation, the vertical intercepts (b) signify the extent of variation range attributed to the patient’s inherent craniofacial condition, while the slopes (k) represent the degree of efficiency in which changes in SN-OP influence alterations in FHR values. Similarly, the simulated strategy was illustrated by Fig. 2B and was also adopted to model FA to elucidate the correlation between FA and SN-OP changes.

### Statistical analysis

Graphpad Prism 9 was used for the statistical evaluations. Normality and lognormality of the data was tested and evaluated by Kolmogorov-Smirnov test. For normally distributed data, test for homogeneity of variance was carried out. One-way ANOVA and Bonferroni test were applied to evaluate the differences between groups. For data which did not show normal distribution, Kruskal-Wallis test and Dunn’s multiple comparisons test were employed for difference analysis. Means and standard deviations were used for comparison. Statistical significance was set at 5%.

## Results

### Dataset construction

After the exclusion of patients with unclear cephalometry images, 1220 of 1301 samples were collected. In order to get a relatively balance sample size of each skeletal type to avoid fitting deviation of the model, we randomly excluded half of the Class I samples considering that it originally had nearly twice the number of Class II and III. After that, 903 samples were included and distribution of samples in different skeletal classes were displayed in Table 3. The overview of cephalometric measurements was listed in Table 4 in the form of means and standard deviations of each skeletal group. Intriguing results could be revealed by the cephalometric data through intra- and inter-group comparison. First, average values of indicators as SN-MP, FMA and S-Go/N-Me for vertical and SNA, SNB and ANB for sagittal classification showed high consistency with the principle that we followed for grouping, validating the reliability of our classification strategy. Second, specialized growth patterns of each skeletal types could be noticed. For crania and maxilla morphology, our results indicated relatively smaller value of Ptm-A, smaller SNA and NA-FH in hypodivergent group, echoing previous studies which suggested that maxilla of this group was generally shorter and posteriorly positioned than the hypodivergent facial type [19, 20]. Sagittally, maxilla of Class II tends to be longer and more protrusive than Class III [19, 20], as larger value of Ptm-A and NA-FH in Class II group could also be revealed by our study in Table 3. From the aspect of mandible morphology, shorter and retrognathic positioning of mandible was also found in hyperdivergent group represented by shorter Go-Po and Go-Co, smaller FH-NPo and SNB. Intriguingly, the discrepancy of mandible size among different sagittal groups mainly existed in the length of Go-Po, while the length of Go-Co showed closer relationship with vertical classification and remained consistent among sagittal types, indicating that mandibular corpus rather than ramus could show higher heterogeneity in different sagittal groups.

**Table 3:** Distribution of samples in various sagittal and vertical skeletal classes

	Hypodivergent		Normodivergent		Hyperdivergent		Total		
	Female	Male	Female	Male	Female	Male	Female	Male	All
**Class I**	62	40	67	42	63	43	192	125	317
**Class II**	52	33	65	42	62	39	179	114	293
**Class III**	62	41	64	39	53	34	179	114	293
**Total**	176	114	196	123	178	116	550	353	
	290	319		294				903

**Table 4:** Comparison of skeletal and dental cephalometric parameters of patients with different skeletal types

	Class I			Class II			Class III			Inter-group Comparison
	Hypo	Nor	Hyper	Hypo	Nor	Hyper	Hypo	Nor	Hyper	
N-ANS	51.9 ± 3.2	52.4 ± 3.5	53 ± 3.3	53.1 ± 4.3	52.5 ± 3	53.1 ± 3.2	52.7 ± 2.9	52.8 ± 3.3	53.5 ± 3.5	N.S
S-N	63.1 ± 3.7	63.2 ± 4.2	63 ± 3.3	64.3 ± 4.1	63.2 ± 4.1	62.7 ± 3.8	64 ± 4.2	63.8 ± 4.1	63 ± 3.8	N.S
Ptm-A	45.5 ± 3.2	44.1 ± 3.5	43.6 ± 3.4^b^	47.6 ± 3.7	46 ± 3.4	45.1 ± 2.8^b^	44.8 ± 3.1	44.1 ± 3.9	42.4 ± 3.2^b^	Hypo:A,C Nor: A,C Hyper: A,C
SNA	82.3 ± 2.7	80.1 ± 2.3^a^	78.6 ± 3.2^b,c^	83.8 ± 2.3	82.2 ± 3.1^a^	80.9 ± 2.8^b,c^	81.6 ± 3.3	80 ± 3.1^a^	77.3 ± 2.9^b,c^	Hypo:A,C Nor: A,C Hyper: A,C
NA-FH	91.6 ± 3	90.8 ± 2.6	89.5 ± 2.4^b,c^	92.2 ± 2.9	92.2 ± 2.5	91.5 ± 3.3	90.1 ± 2.9	89.8 ± 2.9	87.9 ± 2.3^b,c^	Hypo:B,C Nor: A,C Hyper: A,C
PP-FH	0.1 ± 3	0.9 ± 2.6	1 ± 2.8	1 ± 2.5	0.6 ± 2.9	1 ± 3.2	0.9 ± 2.7	0.8 ± 3.5	1.5 ± 3	N.S
Go-Po	72.6 ± 5	71.7 ± 5	70.6 ± 4.5	70 ± 5.2	69.2 ± 4.6	67.9 ± 4.3	75.7 ± 4.6	74.2 ± 5.8	74.2 ± 4.5	Hypo:A,B,C Nor: A,B,C Hyper: A,B,C
Go-Co	59.9 ± 4.3	56.3 ± 4.3^a^	53.2 ± 4.3^b,c^	60.3 ± 5	55.7 ± 3.7^a^	52.3 ± 4.5^b,c^	60.9 ± 5	58.8 ± 4.9	54.6 ± 3.7^b,c^	Nor: B,C
S-Go	79.2 ± 5.8	74.2 ± 5^a^	70.4 ± 5.9^b,c^	79.8 ± 7	73.8 ± 4.3^a^	69.8 ± 5.6^b,c^	80.4 ± 6.3	77 ± 6.2^a^	71.9 ± 4.2^b,c^	Nor: B,C
SN-OP	13.7 ± 3.8	17.8 ± 3^a^	19.9 ± 3.1^b,c^	16.1 ± 3.2	18.4 ± 3.8^a^	21.9 ± 3.5^b,c^	11.2 ± 3.7	14.7 ± 4^a^	17.5 ± 4.2^b,c^	Hypo:A,B,C Nor: B,C Hyper: A,C
S-Go/N-Me	70.1 ± 3	64.4 ± 1^a^	60.4 ± 2.5^b,c^	69.3 ± 2.6	64.3 ± 1.5^a^	59.8 ± 2.7^b,c^	70.8 ± 3.3	65.8 ± 1.7^a^	60.9 ± 2^b,c^	N.S
FH-NPo	89.4 ± 2.7	88.2 ± 2.8^a^	86.7 ± 2.5^b,c^	86.8 ± 2.7	86.3 ± 2.7	84.5 ± 3.5^b,c^	92.2 ± 2.5	91.3 ± 3.2	89.5 ± 2.8^b,c^	Hypo:A,B,C Nor: A,B,C Hyper: A,B,C
FMA	18 ± 3.6	24 ± 3.4^a^	29 ± 3.8^b,c^	21.7 ± 3.5	25.8 ± 3.5^a^	32.1 ± 4.8^b,c^	17.5 ± 3.4	23 ± 3.1^a^	27.6 ± 3.4^b,c^	Hypo:A,C Nor: A,C Hyper: A,C
SN-MP	27.3 ± 3.7	34.7 ± 2.5^a^	40 ± 3.2^b,c^	30.1 ± 3.5	35.8 ± 3.1^a^	42.7 ± 4.3^b,c^	26 ± 3.4	32.8 ± 2.7^a^	38.2 ± 3.6^b,c^	Hypo:A,C Nor: B,C Hyper: A,C
SNB	79.1 ± 2.6	76.9 ± 2.3^a^	75.4 ± 3.3^b,c^	77.7 ± 2.3	76 ± 3.2^a^	74 ± 3^b,c^	82.6 ± 3.7	81 ± 3.6^a^	78.4 ± 3.5^b,c^	Hypo:B,C Nor: B,C Hyper: A,B,C
ANB	3.2 ± 0.9	3.2 ± 1.1	3.2 ± 1.1	6.1 ± 0.8	6.2 ± 1	6.9 ± 1.5	−1 ± 1.9	−0.9 ± 1.9	−1.1 ± 1.8	Hypo:A,B,C Nor: A,B,C Hyper:A,B,

*Intra-group comparison: “a”: significant differences between Hypo- and Nor-group; “b”: significant differences between Hypo- and Hyper-group; “c”: significant differences between Nor- and Hyper-group

*Inter-group comparison: “A”: significant differences between Class I and II group; “B”: significant differences between Class I and III group; “C”: significant differences between Class II and III group

### Performance of the BP neural network

**Fig. 3:**
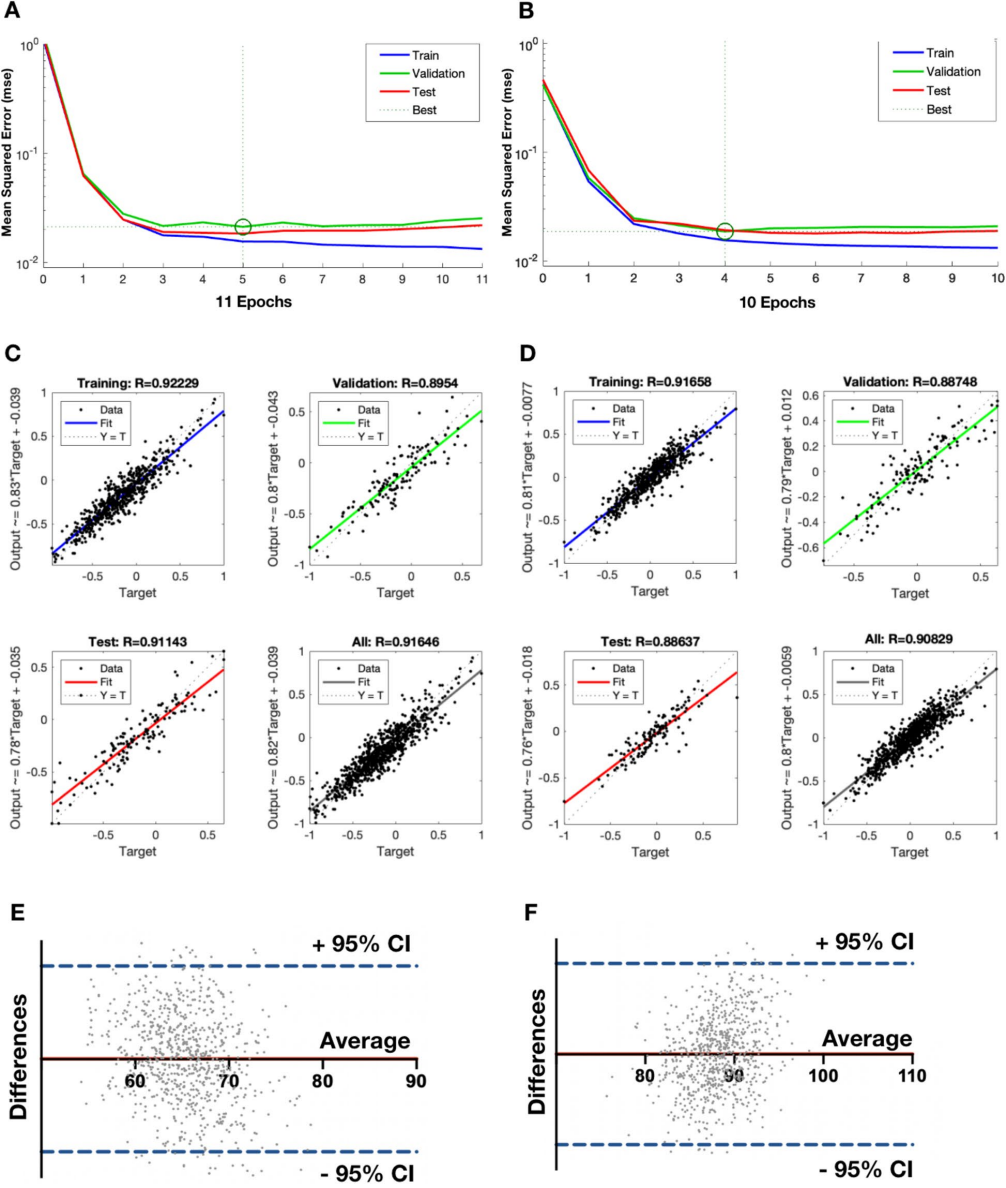
Performance of BP-ANN models. **A**,** B** The relationship curve between the performance loss function of model and iteration times of FHR and FA models respectively. The blue, green and red line represents the cross-entropy over the course of training, validation and test, individually. **C**,** D** Regression analysis of the relativity between predicted and true values of training, validation and test sets of FHR and FA models respectively. **E**,** F** Bland-Altman analysis of pairs of predicted and true values of FHR and FA models respectively. Horizontal axis represents the average value of the pairs and vertical axis represents the difference of the pairs. Red line represents the average of the differences and dotted blue line represent the 95% Confidence interval (95%CI) of the differences

The relationship curve between the performance loss function of the model and iteration training times is shown in Fig. 3A, B, suggesting the training efficacy of the BP-ANN network. The blue, green and red line represents the cross-entropy over the course of training, validation and test, individually. The mean squared error (MSE) of fitting decreased along with the training process which gradually reached convergence during the process of 11 and 10 iterations for FHR model and FA model, respectively. The best performance evaluated by MSE of validation samples was 0.0133, at the 5th iteration for FHR model and 0.0186 at epoch 4 for FA model. Regression coefficients which calculated the relativity between predicted and true values of training, validation and test sets, respectively (Fig. 3C, D), were all above 0.88, representing the accuracy of prediction. For consistency verification, paired-samples *T* test and Bland-Altman analysis were carried out. The paired-samples *T* test’s results indicated that the predicted and actual values were well paired as the correlation coefficient being 0.9007 (*p* < 0.001), while there was no significant difference between predicted and true values as the *t* value was 0.1994 (*p* > 0.05). The results of Bland-Altman analysis indicated that almost all paired values were included in the 95% Confidence interval (95%CI) (Fig. 3E, F), testifying that the BP-ANN models built in this study could predict the value of FHR and FA well once given the basic information of the craniofacial structure.

### Relationship between occlusal plane, face height and facial angle

After ensuring that our model could successfully predict the according values of FHR and FA once given the inputs, we went on to explore the impact of SN-OP changes on FHR and FA. In the model, we separated the variables into SN-OP which representing the rotation, and anatomic parameters which remain constant against the alternation. By this means, we ensured the rationality of the simulation strategy as to solely change SN-OP angle without altering basic geometrical values. In other words, once given the anatomical parameters of one specific patient, we could simulate the FHR and FA changes to OP alternation (Fig. 2B), and the according ratio was then quantified by linear regression analysis. In this way, the predicted correlation between SN-OP and FHR, FA changes could be concluded in the form of vertical intercepts (b) and slopes (k). After running the stimulation for each sample we collected, a list containing patient’s skeletal inputs and corresponding k and b could be obtained. Thus, the general trends of k and b, which reflects the efficacy of responding alternation of FHR or FA to SN-OP changes, could be summarized for each skeletal type (Tables 5 and 6), respectively. The inter- and intra-group comparison results among diversified skeletal groups would be detailed in following sections.

**Table 5:** Predicted correlation between FHR and SN-OP in different skeletal types

		Hypo	Nor	Hyper	Total
Class I	k	− 0.28 ± 0.03	− 0.28 ± 0.04	− 0.28 ± 0.04	− 0.28 ± 0.03
	b	73.07 ± 2.11	70.14 ± 1.69^a^	66.97 ± 2.8^b,c^	70.36 ± 3.22
Class II	k	− 0.27 ± 0.04	−0.28 ± 0.04	− 0.29 ± 0.04^b^	− 0.28 ± 0.04
	b	72.97 ± 1.28	70.12 ± 1.96^a^	67.74 ± 2.82^b,c^	70.82 ± 2.3
Class III	k	−0.26 ± 0.03	−0.28 ± 0.03^a^	− 0.27 ± 0.04	−0.27 ± 0.03
	b	72.61 ± 2.52	70.73 ± 2.33^a^	66.97 ± 1.68^b,c^	70.71 ± 3.27
Total	k	−0.27 ± 0.03	−0.28 ± 0.04	− 0.28 ± 0.04	
	b	73.02 ± 2.06	70.56 ± 1.78	67.64 ± 2.4	
Inter-group comparison	k	N.S	N.S	N.S	
	b	N.S	N.S	N.S	

*Intra-group comparison: “a”: significant differences between Hypo- and Nor-group; “b”: significant differences between Hypo- and Hyper-group; “c”: significant differences between Nor- and Hyper-group

**Table 6:** Predicted correlation between FA and SN-OP in different skeletal types

		Hypo	Nor	Hyper	Total
Class I	k	−0.4 ± 0.07	−0.31 ± 0.09^a^	− 0.25 ± 0.09^b,c^	− 0.33 ± 0.1
	b	95.47 ± 2.25	94.21 ± 2.5	91.45 ± 3.27^b,c^	93.92 ± 3.08
Class II	k	−0.37 ± 0.06	−0.32 ± 0.07^a^	− 0.23 ± 0.08^b,c^	−0.31 ± 0.09
	b	94.42 ± 2.44	93.14 ± 2.57	90.63 ± 3.5^b,c^	92.9 ± 3.15
Class III	k	−0.47 ± 0.07	−0.41 ± 0.08	− 0.34 ± 0.09^b,c^	−0.41 ± 0.1
	b	96.78 ± 2.14	96.07 ± 2.3	94.72 ± 2.41^b^	96.13 ± 2.27
Total	k	−0.41 ± 0.08	−0.34 ± 0.09	− 0.27 ± 0.1	
	b	95.52 ± 2.47	94.41 ± 2.71	92.18 ± 3.6	
Inter-group comparison	k	B,C	B,C	B,C	
	b	C	B,C	B,C	

*Intra-group comparison: “a”: significant differences between Hypo- and Nor-group; “b”: significant differences between Hypo- and Hyper-group; “c”: significant differences between Nor- and Hyper-group

*Inter-group comparison: “A”: significant differences between Class I and II group; “B”: significant differences between Class I and III group; “C”: significant differences between Class II and III group

### k of FHR to SN-OP

**Fig. 4:**
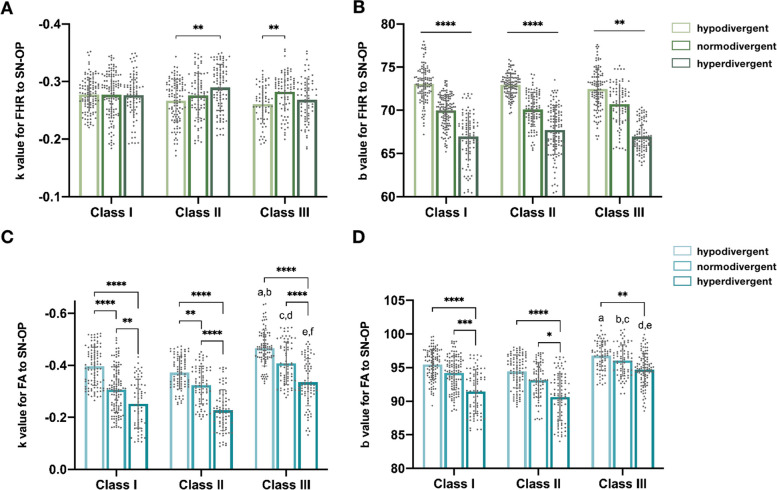
Comparison of the predicted correlation between SN-OP and FHR, FA respectively in different skeletal types. **A** Comparison of slopes for FHR to SN-OP. **B** Comparison of vertical intercept for FHR to SN-OP. **C** Comparison of slopes for FA to SN-OP. a: *P* < 0.05 vs. I-hypo group, b: *P* < 0.01 vs. II-hypo group, c: *P* < 0.0001 vs. I-nor group, d: *P* < 0.0001 vs. II-nor group, e: *P* < 0.0001 vs. I-hyper group, f: *P* < 0.0001 vs. II-hyper group. **B** Comparison of vertical intercept for FHR to SN-OP. a: *P* < 0.01 vs. II-hypo group, b: *P* < 0.01 vs. I-nor group, c: *P* < 0.0001 vs. II-nor group, d: *P* < 0.0001 vs. I-hyper group, e: *P* < 0.0001 vs. II-hyper group

In the linear regression analysis of FHR, the slopes (k) represented the changing rate of FHR to SN-OP. Clinically, larger k meant more sensitive change of FHR to OP rotation. However, k in all skeletal types appeared to be constant and only showed significant difference as k^II-hypo^ < k^II-hyper^ (*p* < 0.01), k^III-hypo^ < k^III-nor^ (*p* < 0.01) (Fig. 4A). The average slope of all samples was about − 0.28 ± 0.05, indicating that by increasing 10 degree of OP angle, the predicted decrease of FHR was about 2.8%.

### B of FHR to SN-OP

On the other hand, the vertical intercepts (b) representing the range of FHR decided by skeletal types, showed great divergence among different vertical skeletal types (Fig. 4B). Significantly, the decline tendency as b^hypo^ > b^nor^ > b^hyper^ was similar in every sagittal type (*p* < 0.01), consistent with the fact that hypodivergent type usually has shorter face and higher FHR. The average value of b in hypo-, nor-, hyper-groups are about 73, 71 and 68, while the average slope (k) of SN-OP of each group are 14, 17, 20 (Table 3). Combined with the function of regression as FHR = b + k × SN-OP, the average outcome of FHR could be around 69, 65 and 61, which highly echoed the normative range and classification basis of FHR for each vertical group, conforming the reliability of our models.

### K of FA to SN-OP

As for FA prediction, the change rate appeared to be discrepant among skeletal types (*p* < 0.01), but with some regularity to conform. In summary, hypodivergent group and Class III group, tended to have higher |k| in vertical and sagittal comparison, respectively. Vertically, the slopes (k) showed consistent decline trend as k^hypo^ > k^nor^ > k^hyper^ inside every sagittal type (Fig. 4C). Significant differences between each pair in Class I and Class II were also testified. As for Class III, the discrepancy was significant as k^III-hypo^ > k^III-hyper^ (*p* < 0.0001) and k^III-nor^ > k^III-hyper^ (*p* < 0.0001), but not between k^III-hypo^ and k^III-nor^ (*p* > 0.05). Furthermore, the sagittal classification also matters. Though classified as the same vertical types, the k of Class III appeared to be larger than that of Class I or II in all vertical groups (Fig. 4C, a-f, *p* < 0.05), while there is no significant difference of k between Class I or II in each vertical groups (*p* > 0.05).

### B of FA to SN-OP

Similarly, the vertical intercepts (b) reflected the tendency of convexity (Fig. 4D), which was largely decided by the skeletal structure. Generally, hyperdivergent and Class II group showed higher tendency to have more convex profile and lower scores of FA. Intriguingly, our results also validated significant differences of FA-b between hypo- and hyper- groups and between Class II and III. In details, a decline tendency as b^hypo^ > b^nor^ > b^hyper^ was shown inside every sagittal group. Significant differences between b^hypo^ and b^hyper^ existed in Class I (*p* < 0.0001), II (*p* < 0.0001) and III (*p* < 0.01), while only Class I (*p* < 0.001) and II (*p* < 0.05) showed significant differences by comparing b^nor^ and b^hyper^. No significant divergences were found between b^hypo^ and b^nor^ in neither Class I, II or III (*p* > 0.05). For sagittal comparison, discrepancy as b^III^ > b^II^ was validated in each vertical groups (*p* < 0.0001). Significantly b^III^ > b^I^ was found in normodivergent group (*p* < 0.01) and hyperdivergent group (*p* < 0.0001), but not in hypodivergent group (*p* > 0.05). No significant difference between b^I^ and b^II^ was found in each vertical groups (*p* > 0.05). As for the clinical meaning of the b value, similar calculation as FA = b + k × SN-OP could be done as for FHR. By the mathematical function, the average values could be calculated which corroborated with the facts that hypodivergent Class III patients tend to have concave face (FA > 90°), while hyperdivergent Class II patients are more apt to convex profiles (FA < 85°). Accordingly, the consistency of our function with the reality knowledge also verified the reliability of our models.

## Discussion

Artificial intelligence and machine learning, standing as landmark invention of twenty-first century, has gained reputation in both clinical application [21] and scientific research of modern dentistry [22]. Among diversified strategy of machine learning, ANN-BP [23] could be an optimal choice for statistical tasks characterized by perplex interactions among variables and involving mass data where traditional mathematics analysis could fail. It could make the utmost of data without pre-filtering by human, which avoid subjective bias as mankind clinical practitioner could usually have. Meanwhile, ANN-BP model already has its efficiency proved in clinical use, as to predict facial deformation after complete denture restoration [24] as well as the prediction of pathologic index [25]. Just as in these cases, practitioners typically rely on their clinical experiences to make prediction about facial improvement qualitatively against OP angle changes without statistical support. However, considering the interconnected anatomical and geometric relationship of cranium, maxilla and mandible, a complex non-linear function could possibly exist among the structures, which could then be learned by machine as our ANN-BP model could successfully predict the FHR and FA once given the anatomical skeletal parameters and angle of SN-OP. Compared to linear regression model which is typically used for predictive analysis in orthodontists, ANN-BP models could be more suitable for hypothesis where variables are interdependent and have interaction relationship. Meanwhile, its self-learning ability makes it unnecessary for researchers to presuppose a model structure which could largely lead to objective bias. Additionally, through repeated training and massive data processing, it could be equipped with adequate robustness against measuring error to a certain degree, which could be common in cephalometric analysis [26, 27].

In choosing a BP-ANN over a Convolutional Neural Network (CNN) for this study, the decision was guided by the nature of the data and research goals. Cephalometric data, comprising numerical orthodontic measurements, aligns with BP-ANN’s strength in handling structured numerical information, whereas CNNs are more adept at processing grid-like data, such as images. Given the study’s primary objective of predicting relationships between craniofacial parameters and facial aesthetics, the regression-focused capabilities of BP-ANN were deemed more suitable than the image-related tasks typically associated with CNNs. Additionally, the straightforward structure of cephalometric data rendered the complex feature extraction capabilities of CNNs unnecessary, as the data is preprocessed and represented as numerical features. Therefore, for tasks involving structured numerical data and regression modeling, BP-ANN emerged as the more appropriate choice in this context.

The success of any predictive model hinges on the logical connections among the chosen variables, and ours was rooted in the principles of gnathology. Generally, the key to healthy gnathology system is the balance among three elements as teeth, bones and muscles [28], representing by OP, skeletal structures of nasomaxillofacial and mandibular complexes, lower facial height, respectively. While orthodontic therapy for adults could merely change anatomical parameters as cranio-maxilla angles and length, the remaining two factors as OP and lower facial height could be inter-connected, explaining the possibility of FHR and FA prediction based on OP and skeletal factors. Previous studies have indicated that greater hyperdivergence and a smaller anteroposterior jaw discrepancy result in a larger angle between OP and FH [29], which was also supported by our statistical results of OP-SN in diversified skeletal types.

**Fig. 5:**
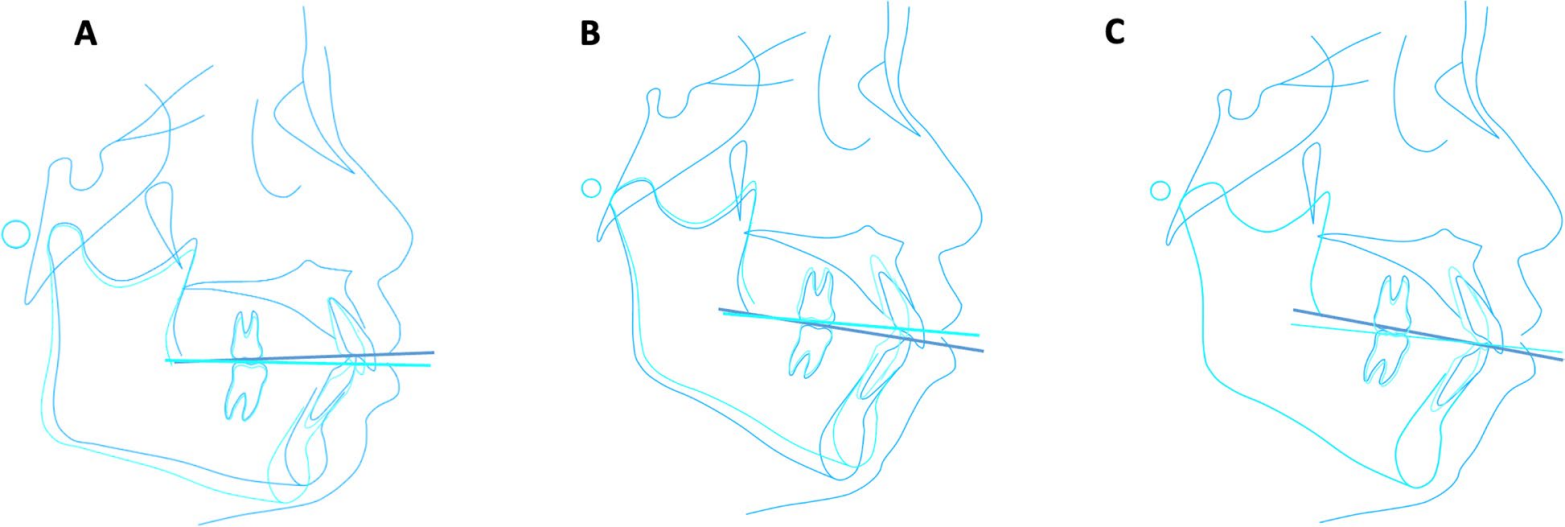
Schematic illustration of the SN-OP changes and consequent mandibular rotation. (**a**) Clockwise rotation of OP and mandible by extrusion of upper and lower molars and incisors in hypodivergent group. (**b**) Counter-clockwise rotation of OP and mandible by intrusion of upper and lower molars and incisors in hyperdivergent group. (**c**) Counter-clockwise rotation of OP without beneficial mandible rotation by extrusion of upper molars, intrusion of lower molars and incisors in hyperdivergent group

For orthodontists, the OP control during treatment was in essence vertical control, which could be concluded by three main strategies as mesial and distal molar movement, vertical control of the maxillary and mandibular molars, and extrusion and intrusion of incisors. The consequent rotation of mandible, is essentially the adaption to new occlusion. Our study has revealed divergent efficiency in how FA and FHR change in response to SN-OP among different skeletal types. Several explanations exist:

1. Different rotation radius: Geometrically, rotation with larger radius could lead to larger distance changes within the same angle. Thus, mandible length as hypo- > nor- > hyperdivergent group, and Class III > I > II could partially explain the results as higher efficiency in FA changes of hypo- and Class III group. However, since FHR equals the ratio of N-Me/S-Go and groups with larger mandible also displayed larger S-Go, the radius length effect could be offset to some degree, which result in a relatively consistent k-FHR.
2. Different orthodontic strategies to change SN-OP: For hypodivergent group, extrusion of molars and clockwise rotation of incisal occlusal point could be the most efficient method to realize mandible clockwise rotation responding to SN-OP changes (Fig. 5A). Conversely, in hyperdivergent group, the most effective way for mandible rotation is by intrusion of molars and counterclockwise rotation of incisal occlusal point (Fig. 5B), which means great intrusion of incisors. However, bodily intrusion or extrusion along the axis is physiologically limited, leading to root resorption concerns [30, 31]. Meanwhile, relative porous bone of hyperdivergent patients made it easier to lose the vertical anchorage resulting extrusion of molars. Under this situation, even though anterior tooth intrusion has already realized, OP could change without beneficial mandible rotation (Fig. 5C). Conclusively, the ideal method to realize maximal mandible rotation to SN-OP change could be intractable and limited for hyperdivergent group, which explains the lower k-FA. For hypodivergent group, on the other side, patients usually have deep overbite and the clockwise rotation of incisal occlusal point could be easy to achieve even without much extrusion of incisors, thus higher k-FA.

From a therapeutic perspective, our findings offer valuable clinical insights. For hypodivergent groups, as FA is more sensitive to OP changes, clinicians can leverage this to enhance profile improvements for Hypo-Class III patients. However, caution should be exercised with Hypo-Class II patients, as clockwise rotation can benefit Class III profiles but potentially harm Class II profiles. For Hypo-Class III patients, adequate extrusion of molars and minimal intrusion of anterior tooth could be suggestive for maximization FA changes. On the contrary, less molar movement and more intrusion of anterior tooth during leveling could be beneficial for Hypo-Class II patients’ profile. For hyperdivergent group, if more effective counterclockwise rotation of mandible is needed during treatment, more attention should be put on the control of vertical anchorage control of molars and the feasibility of long-distance intrusion of anterior tooth, since FA is revealed to be more difficult to change during OP rotation.

## Conclusions

Based on the theoretical, geometrical and clinical significance, we trained 2 BP-ANN models to predict the profile improvement induced by OP angulation under specific skeletal parameters. The rates of FHR changes to SN-OP appeared to be constant among groups as 10 degrees of SN-OP changes could lead to about 2.8% change of FHR. In FA models, SN-OP rotation tended to be more efficacy in resulting facial angle changes in hypodivergent group and Class III group. By taking advantage of the modeling ability of AI confronting non-linear problems and interdependent variables, we hope to provide orthodontists a quantitative evidence of the efficiency of SN-OP changes to aesthetic improvement as a reference.

## Data Availability

The datasets generated and analyzed during the current study are not publicly available due to patients’ privacy but are available from the corresponding author on reasonable request.

## Abbreviations

OP: Occlusal plane
BOP: Bisected occlusal plane
FOP: Functional occlusal plane
BP-ANN: Back-propagation artificial neural network model
ICC: Intra-class Correlation Coefficient
FHR: P-A face height ratio
FA: Facial angle
MSE: Mean squared error
